# Clinicians’ Perceptions of Nature-Based Interventions in Mental Health Care: A Qualitative Descriptive Study from Pakistan

**DOI:** 10.3390/bs16081298

**Published:** 2026-07-30

**Authors:** Yumna Ali, Akihiko Ozaki, Yasuhiro Kotera

**Affiliations:** 1 Department of Psychology, Hazara University, Mansehra 21120, Pakistan; 2 Medical Governance Research Institute, Tokyo 108-0074, Japan; 3 Breast and Thyroid Center, Jyoban Hospital of Tokiwa Foundation, Iwaki 972-8322, Japan; 4 Faculty of Medicine and Health Sciences, University of Nottingham, Nottingham NG7 2RD, UK; 5 Center for Infectious Disease Education and Research, Osaka University, Osaka 565-0871, Japan; 6 Department of Social Sciences, Azerbaijan University, AZ1007 Baku, Azerbaijan

**Keywords:** nature-based interventions, mental health, clinicians, green space, qualitative research, Hazara, Pakistan

## Abstract

Background: Mental health conditions remain a major yet underrepresented public health concern in Pakistan and globally, highlighting the need for innovative and complementary interventions. Nature-based interventions (NBIs), which involve structured engagement with natural environments to enhance health and wellbeing, offer a promising approach, particularly in the Hazara Division of Khyber Pakhtunkhwa, Pakistan, a region renowned for its Himalayan forests, scenic valleys, and rich biodiversity. Methods: This study employed a qualitative descriptive design to explore mental health clinicians’ perceptions of NBIs in community mental health settings. Fourteen clinicians from the Hazara Division, Khyber Pakhtunkhwa, Pakistan, were recruited using purposive and snowball sampling between February and March 2026. Data were collected through semi-structured interviews and audio-recorded. Transcripts were analysed using inductive thematic analysis following Braun and Clarke’s framework, supported by HyperRESEARCH software. Results: Participants generally perceived engagement with nature as calming, restorative, and therapeutically beneficial, and many reported informally encouraging clients to participate in nature-related activities. Clinicians expressed willingness to integrate NBIs into routine practice and identified potential benefits including improved mood, relaxation, empowerment, and enhanced social connectedness. However, several barriers were also identified, including client-related factors such as anxiety, low motivation, and skepticism, as well as contextual and organisational constraints such as gender-based limitations in access to natural spaces and safety-related policies. Conclusions: Despite these challenges, clinicians demonstrated strong support for NBIs, suggesting their potential as a feasible adjunct to mental health care in Pakistan. Addressing identified barriers may facilitate integration into routine services and enhance access to holistic mental health support in community settings.

## 1. Introduction

Mental health disorders remain a major global public health challenge and a leading cause of disability and reduced quality of life. In Pakistan, access to mental healthcare is particularly limited, especially for the nearly 60% of the population living in rural areas where specialist services are scarce (Choudhry et al., 2023; Dayani et al., 2024). More than 15 million people are estimated to experience mental health disorders despite the availability of only around 400 psychiatrists nationwide (Javed et al., 2020).

Depressive disorders account for a substantial proportion of disability-adjusted life years (DALYs), while underreporting and inadequate surveillance continue to underestimate the true burden of mental illness (Alvi et al., 2024; Mahesar et al., 2025). These challenges underscore the need for accessible, culturally appropriate, and cost-effective interventions. Healthcare professionals are also frequently exposed to occupational stress and psychological distress, increasing the need for accessible and sustainable mental health interventions (Abbaspour et al., 2020).

Nature-based interventions (NBIs) have emerged as promising and economically sustainable approaches, particularly in biodiversity-rich low- and middle-income countries (LMICs) (Vicarelli et al., 2024).

Growing evidence suggests that non-pharmacological interventions can effectively complement conventional mental healthcare for individuals experiencing cognitive and affective symptoms (McWhirter et al., 2020; Wijeratne et al., 2022). Among these, NBIs consistently improve symptoms of depression, anxiety, stress, and psychological well-being (Kotera et al., 2020). Activities such as forest bathing and walking in natural environments reduce psychological distress, rumination, stress, and enhance mood and physical health (Chen et al., 2025; J. Ma et al., 2024). Their benefits are attributed to multiple pathways, including exposure to natural environments, increased physical activity, and enhanced social connectedness (Kang et al., 2023; Madsen et al., 2026), while sustained exposure appears more effective than short-term engagement in reducing depressive symptoms (Jessen et al., 2025).

In this study, NBIs are defined as intentional, structured or semi-structured activities that engage individuals with natural environments to promote physical, psychological, and social well-being. They include nature-based mindfulness and ecotherapy (Burger et al., 2024), therapeutic horticulture and healing gardens (Catissi et al., 2024), nature savoring practices (Charisi et al., 2025), green exercise (Gritzka et al., 2020), forest bathing (Kotera, 2025), wilderness therapy (Kaleta et al., 2025), and social or clinical nature prescriptions (Menhas et al., 2024). Across clinical and community populations, NBIs have been associated with improvements in depression, anxiety, stress, emotional regulation, cognitive functioning, attentional restoration, and overall psychological well-being (Johansson et al., 2022; Kotera & Fido, 2022; Moll et al., 2022; Paredes-Céspedes et al., 2024; Silva et al., 2023; Vella-Brodrick & Gilowska, 2022; Vitale & Bonaiuto, 2024). Group-based programmes may further reduce loneliness, strengthen social connectedness, enhance community resilience, and contribute to wider socioeconomic benefits (Chausson et al., 2024).

The successful implementation of NBIs increasingly depends on clinicians, who facilitate nature prescriptions by encouraging engagement with natural environments alongside conventional care (Nguyen et al., 2023). Green prescriptions have been adopted by psychologists, psychiatrists, general practitioners, and other healthcare providers for individuals experiencing stress, emotional distress, or sedentary lifestyles (Thomson et al., 2020; Wood et al., 2022). Their successful integration into healthcare systems requires clinicians’ support, favourable organisational environments, and multidisciplinary collaboration (Tambyah et al., 2022; Tate et al., 2024). International initiatives, including multidisciplinary programmes for postnatal mental health and the Scottish Green Health Prescription pathway, demonstrate the feasibility of integrating NBIs into routine care (Hall et al., 2023; Marx & More, 2022). Nevertheless, heavy workloads, burnout, and limited organisational resources continue to impede implementation (Heimlich-McQuarters et al., 2026; Rosanne et al., 2024).

Despite increasing international evidence, research on NBIs has been conducted predominantly in Western and East Asian settings, with limited evidence from South Asia and other LMICs. Pakistan, particularly the Hazara Division of Khyber Pakhtunkhwa, provides a distinctive socioecological setting characterised by Himalayan landscapes, forests, rivers, and strong cultural connections with nature (Haq et al., 2025). The region, home to more than six million people, continues to experience substantial mental health challenges following the 2005 earthquake, compounded by poverty, restricted healthcare access, and limited specialist mental health services (Anwar et al., 2011; Commissioner Hazara Division, n.d.; Sana & Nath, 2017). Mental healthcare is delivered through the WHO Mental Health Action Plan and Pakistan’s Comprehensive Mental Health Services Plan, with community services supported by the adapted Mental Health Gap Action Programme Humanitarian Intervention Guide (Gilani & Shah, 2022; Humayun & Najmussaqib, 2025). Hazara’s forests, rivers, glacial lakes, and mountainous landscapes provide a favourable environment for implementing NBIs, although urbanisation and climate-related pressures highlight the need for sustainable green infrastructure (Batool et al., 2025; Rayan et al., 2022).

Unlike settings where contact with nature is primarily recreational, interaction with natural environments in Hazara is embedded within everyday social and cultural life. However, evidence regarding clinicians’ perceptions of NBIs in South Asian LMICs remains scarce. Accordingly, this study investigates clinicians’ perceptions of nature-based interventions in the Hazara Division of Pakistan, examining how environmental familiarity, cultural norms, and healthcare system constraints influence their acceptance and implementation (Ali et al., 2026). By addressing this important geographical and cultural gap, the study extends the evidence base for NBIs in LMICs and informs the integration of nature-based mental healthcare into resource-constrained health systems.

## 2. Materials and Methods

### 2.1. Study Setting and Design

This study used a qualitative descriptive design to provide a clear, data-close account of participants’ experiences and perspectives. The design informed the use of semi-structured interviews and inductive thematic analysis, ensuring that findings remained closely grounded in participants’ accounts with minimal interpretation (Sandelowski, 2000).

The study followed the qualitative descriptive framework proposed by Doyle et al. (2020). Semi-structured interviews were conducted with mental health clinicians working in Hazara Division, Khyber Pakhtunkhwa, Pakistan, to explore their perceptions of implementing NBIs within community mental health services. This checklist has been completed in accordance with the COREQ (Consolidated Criteria for Reporting Qualitative Research) guidelines to ensure transparency and rigor in reporting qualitative findings (see Supplementary File S2: COREQ 32-item Checklist). To ensure confidentiality in a small and potentially identifiable professional sample, quotations are identified using participant numbers rather than detailed demographic descriptors. The interview guide was developed collaboratively by the research team. Although it was not formally pilot-tested, it was applied flexibly and refined iteratively during early interviews to improve clarity and depth of questioning, while maintaining consistency across the data collection process (See: Supplementary File S1: Interview Guide for Mental Health Clinicians in Hazara Division).

Throughout this study, the term patient refers to individuals accessing mental health services, reflecting the terminology commonly used within the local healthcare system (Javed et al., 2020). Ethical approval was obtained from Hazara University, Khyber Pakhtunkhwa, Pakistan.

### 2.2. Recruitment and Sample Characteristics

Participants were recruited through purposive sampling from mental health services across the Hazara Division, as shown in Table 1. Eligible participants were required to be aged 18 years or older and currently involved in providing direct mental health support within the region. Recruitment notices were distributed to mental health staff via organisational email lists by a Mental Health Support Worker and a Social Support Worker on behalf of the research team. Snowball sampling was subsequently used, whereby participants referred other potentially interested clinicians to the researchers. This approach resulted in the recruitment of four additional participants who had not initially been reached through formal recruitment channels.

Consistent with qualitative research standards, sample adequacy was determined through information power and thematic saturation rather than statistical representativeness. Participants were purposively selected for their direct relevance to the research question. Sample adequacy was guided by information power and thematic saturation rather than statistical representativeness. Data collection ceased after 14 interviews because ongoing analysis indicated that no new codes, themes, or meaningful insights relevant to the study aims were emerging, and subsequent interviews confirmed repetition of existing patterns. A sample can be smaller if it holds dense, highly relevant information; size depends on study aim, sample specificity, use of theory, dialogue quality, and analysis strategy (Wutich et al., 2024). Several authors recommend using information power, conceptual depth, or theoretical sufficiency to judge data adequacy rather than positivist notions of “enough” cases (Naeem et al., 2024). Overall, the sample reflected the multidisciplinary nature of the mental health workforce within the Hazara Division.

Of the 14 mental health clinicians interviewed, five reported actively implementing nature-based interventions (NBIs) in their clinical practice. The remaining nine clinicians had not yet used NBIs but shared their perspectives regarding their perceived effectiveness, implementation barriers and facilitators, and their willingness to incorporate NBIs into future practice.

### 2.3. Data Collection

Data were collected between February and March 2026 through semi-structured interviews exploring clinicians’ perceptions of the benefits, opportunities, and barriers to implementing NBIs within mental health services. An interview guide, developed collaboratively by the research team, included open-ended questions such as: “To what extent might the mental health patients you work with benefit from participating in nature walk groups in the short and long term?” and “What do you think may be barriers to using nature walk groups for mental health improvement?” Additional prompts were used to elicit detailed responses. Interviews were conducted individually at locations convenient to participants, lasted 20–68 min (mean = 35 min), and were audio-recorded and transcribed verbatim. Transcriptions were checked against the original audio recordings for accuracy. Member checking was not formally undertaken. An audit trail was maintained through documentation of coding decisions, theme development, and iterative discussions within the research team, supporting transparency and dependability of the analytic process.

The first author-maintained field notes to document contextual observations and reflections. Regular online discussions among the research team supported reflection on the interview process and refinement of interview questions, enhancing the quality and consistency of data collection (Houghton et al., 2013).

Field notes were written immediately following each interview rather than during the interviews. The first author recorded brief reflective notes directly after each session, documenting contextual observations, non-verbal cues, and initial analytic reflections. This approach was used to ensure that note-taking did not disrupt participant engagement during the interviews while still capturing important contextual information while memory was fresh (Phillippi & Lauderdale, 2018).

### 2.4. Data Analysis

An inductive thematic analysis approach was used to analyse the data (Braun et al., 2019). Transcripts were coded and managed using HyperRESEARCH software. The first author followed Braun and colleagues’ six-stage process, beginning with repeated reading for familiarisation, followed by initial coding, and the development, refinement, and synthesis of codes into broader themes representing participants’ perspectives. Reflective field notes supported interpretation and theme development. Credibility was strengthened through regular peer debriefing and online team discussions of coding decisions and interpretations, ensuring interpretive validity consistent with qualitative descriptive methodology (Houghton et al., 2013; Sandelowski, 2000). The first author, a doctoral psychology student from the Hazara region, conducted the study, which facilitated contextual understanding and rapport but also required reflexivity regarding potential assumptions. Reflexivity was maintained throughout the research process. Before data collection, the first author reflected on their positionality as a researcher familiar with the Hazara region and considered how this familiarity might shape assumptions and interpretations. During interviews, efforts were made to remain open and participant-led, using probing questions to explore meanings without imposing prior assumptions. Throughout data collection and analysis, reflective notes were maintained and discussed within the research team to identify and critically examine potential biases, supporting an ongoing reflexive awareness of the researchers’ influence on data generation and interpretation.

The second and third authors, social science academics, supervised the research process; the third author also has expertise in nature therapy (Shinrin-Yoku) and initiated the collaboration. To minimise bias, the team engaged in ongoing reflexive discussions and resolved differing interpretations through consensus. To support transferability, detailed descriptions of the study setting, participants, and procedures are provided (Firestone, 1993).

## 3. Results

Thematic analysis was conducted using the HyperRESEARCH software. Initial coding was performed inductively through repeated reading of transcripts and line-by-line coding. Codes were organised into an evolving coding structure, which informed the development of broader themes. Although analysis was primarily inductive, it was guided by the study aim and refined through iterative discussions within the research team. Preliminary codes and themes were reviewed collaboratively to enhance consistency and interpretive rigour, and final themes were agreed through consensus.

**Figure 1:**
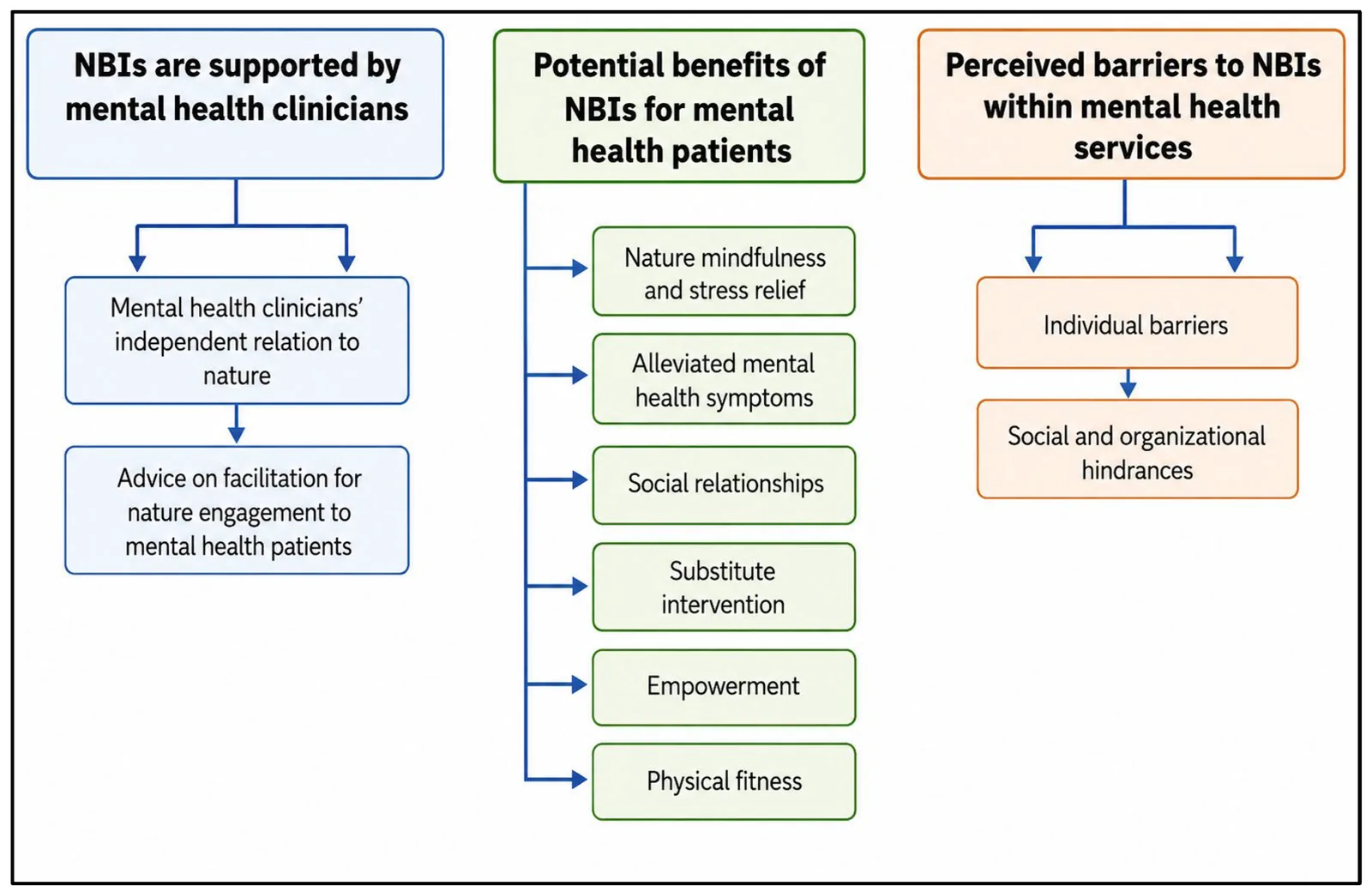
Summary of findings.

Three major themes emerged in the interviews: (1) NBIs are supported by mental health clinicians; (2) potential benefits of NBIs for mental health patients; and (3) perceived barriers to NBIs within mental health services. The anonymity of participants was ensured, and they were referred to as “clinicians” in the results. and any comments included as an example are identified by a number rather than details that risk their anonymity (e.g., age, gender, role). Figure 1 presents the summary of findings, illustrating the full analytic hierarchy. The analysis comprises three overarching themes: Theme 1 includes two subthemes, Theme 2 includes six subthemes, and Theme 3 includes two subthemes.

### 3.1. NBIs Are Supported by Mental Health Clinicians

Clinicians’ support for NBIs was shaped by strong biographical and environmental familiarity with natural landscapes in the Hazara Division. When asked about their own experiences of nature and its influence on wellbeing, many participants described a personal appreciation for natural environments, noting that being in nature fostered feelings of relief, relaxation, and enduring therapeutic benefit. Reflecting this personal connection, several clinicians reported actively encouraging patients to spend time in natural settings as part of their broader therapeutic advice and informal clinical recommendations.

### 3.2. Mental Health Clinicians’ Independent Relation to Nature

Many clinicians described their personal experiences of engaging with nature and exploring natural environments. One participant reflected:

“When we were young, we could grow near the mountainous ranges. Bush walking was common, and so was regular walking. Agroforestry was also the norm, so I would go around the forest to look out for different flora and fauna. It has been a part of life here.”

(14)

These accounts highlight how participants were often raised in proximity to natural landscapes, including forests, mountains, lakes, and rivers. As a result, spending time in nature was described as a familiar and almost routine aspect of life for many clinicians. More than half of the participants reported that engaging in physical activity in natural settings, such as walking or hiking, was a regular practice that also supported their mental well-being.

Several clinicians emphasised the restorative effect of nature on their own mental health. One participant explained:

“Being in nature was supportive for my own well-being. It helps me to relieve stress. When there is a hectic day of work around the clinic, this is beneficial for me to rest.”

(13)

Similarly, another clinician noted that “it relieves stress completely” (10) and frequently recommends time in nature to others. This participant also suggested that exposure to natural environments may support higher-order thinking and cognitive clarity. In addition, some clinicians noted that limited exposure to nature can contribute to increased stress levels.

Another participant described structured engagement with nature as particularly beneficial, stating that a planned routine of spending time outdoors is “healing to remorse, stress, and negative emotions” (08), especially given the demanding and stressful nature of clinical work. Clinicians also reported experiencing feelings of calm and sensory pleasure in natural environments, including appreciation of sounds, smells, and scenic views.

### 3.3. Advice on Facilitation for Nature Engagement to Mental Health Patients

Clinicians strongly supported encouraging patients to engage in NBIs, such as walking and other outdoor activities, and many viewed these activities as being as important as medication in promoting recovery. They emphasised that spending time in nature contributes to more holistic health outcomes. One clinician stated, “nature will always continue to exist as part of the topography” (02), highlighting the enduring and accessible presence of natural environments.

When further discussing NBIs, several participants noted that engaging with nature can be more convenient and less restrictive than structured, office-based therapeutic approaches. They also highlighted the affordability of nature-based activities as a key advantage, particularly in resource-constrained settings. One clinician explained:

“… since we live in a naturally endowed area, engaging and being in nature is so easy. Whether it is about sitting near the river lake for fishing, or walking down trails or mountain seeing, it is fairly easy and at a negligible cost”.

(11)

Some clinicians further suggested that NBIs may strengthen therapeutic relationships and improve clinical engagement. One participant noted that “it is a better way to establish clinical relationships” (08), indicating that shared outdoor experiences may enhance rapport and trust between clinicians and patients. A few clinicians also reported already incorporating natural environments into their clinical practice and assessment processes.

In some instances, clinicians described deliberately shifting therapeutic interactions into natural settings to support client progress. For example:

“I came across a tough client, who was complicated with trauma, yet I took him to the botanical gardens. We could feed the squirrels. We strolled, and then it became an effective meeting place. I could see significant improvement thereon”.

(11)

### 3.4. Potential Benefits of NBIs for Mental Health Patients

Mental health clinicians identified a range of perceived benefits associated with NBIs, including enhanced mindfulness and stress reduction, improvement in mental health symptoms, strengthened social relationships, increased feelings of empowerment, and opportunities for physical activity. Collectively, these outcomes were seen as contributing to improved overall health and well-being.

### 3.5. Nature Mindfulness and Stress Relief

Mental health clinicians described NBIs as offering opportunities for mindfulness, stress reduction, and enriched sensory experiences that support restoration and wellbeing. One clinician elaborated on these perceived benefits as follows:

“… it is important to stay alert regarding the environment that surrounds me, whether it is about appreciating flowers or the birds. It is a meditative, serene experience, and I feel that people would benefit from the calm. You become part of the natural environment and a part of the world that is peaceful, especially in a frantic ecosphere. It enhances social awareness.”

(01)

Nature was therefore seen as enabling individuals to move beyond their internal focus and foster a sense of connection and belonging within a broader, more peaceful environment. Clinicians also emphasised the value of heightened sensory engagement within NBIs. Many participants described specific sensory experiences, such as “feeling grounded while walking on grass with bare feet (03),” active noticing “wood pigeons in the trees (04),” experiencing “the smell of conifers (06),” and handling “wild almonds in my hands (07).”

These sensory encounters were described as calming and restorative, helping to ease feelings of resentment, stress, and demotivation, while also fostering emotions of contentment, freedom, and felicity. Clinicians further suggested that when NBIs are facilitated through structured and guided approaches, such as nature mindfulness programmes, the therapeutic benefits may be strengthened and more consistently realised.

### 3.6. Alleviated Mental Health Symptoms

Mental health clinicians believed that NBIs could offer both short-term and long-term benefits, particularly in relation to anxiety and depression. They described nature walks as potentially supporting physiological regulation, including hormonal balance and the release of endorphins, which in turn may enhance mood and increase feelings of happiness.

One clinician observed:

“The people enjoy the walk, and differences are seen in people. In the mornings, they can move in nature, and they come back different and energized. They smile and feel relieved.”

(10)

Some participants further explained that NBIs may help patients experience relief from internal psychological distress by shifting attention away from inner turmoil toward the external natural environment. One clinician noted that these experiences can create a sense of mental clarity and grounding:

“It also helps to navigate properly and put in words how they feel and express, and link to how their body feels”.

(07)

### 3.7. Social Relationships

Clinicians also believed that NBIs could foster social connection and a sense of belonging, which are considered important components of mental health recovery. They suggested that group-based NBIs may help reduce loneliness and counter patterns of social withdrawal commonly observed among mental health service users. One clinician noted, “nature walking is the only interaction for us” (01), highlighting the limited opportunities for social engagement in some patients’ lives.

Participants emphasised that such interventions should not place pressure on individuals to actively interact if they are not ready. Instead, simply sharing the same space in nature may provide a sense of presence and comfort, allowing participants to gradually become more comfortable with others over time. Clinicians also suggested that informal interaction may emerge more naturally in outdoor settings, where patients can engage in casual conversation without the intensity or pressure of sustained eye contact often experienced in traditional clinical environments.

One clinician explained that walking together “creates a natural flow of conversation that is distinct as compared to a confined sitting space” (09). Others noted that group-based NBIs may also facilitate connections between like-minded individuals, allowing participants to support and motivate one another in their recovery journeys. In this way, clinicians felt that NBIs could help reduce feelings of isolation, reinforce a sense of shared experience, and contribute to the reduction of stigma associated with mental health conditions.

### 3.8. Substitute Intervention

Mental health clinicians described NBIs as a complementary alternative to conventional clinical approaches, particularly for individuals who experience difficulties with medication adherence, home confinement, or the structured nature of sit-down therapy sessions. Some clinicians suggested, based on their clinical experience, that NBIs may be especially beneficial for adolescents, who they perceived as potentially more resistant to traditional interventions and may experience challenges with emotional regulation in formal therapeutic setting.

One clinician explained:

“I think that clinical barriers are eased when another medical practitioner is not staring to expect an answer from the mental health patients.”

(05)

In this context, NBIs were seen as reducing the sense of pressure often experienced in clinical encounters and alleviating the feeling of being “stuck in a room” during therapy sessions. Instead, outdoor environments were described as fostering a more relaxed and supportive atmosphere conducive to mental well-being. The same clinician further noted that NBIs contribute to “an environment that is more supportive of mental health” (05).

### 3.9. Empowerment

Mental health clinicians viewed NBIs, such as nature walking, as beneficial for patients experiencing cognitive and functional difficulties, with additional potential to support empowerment and personal agency. They suggested that as mental health patients participate in these activities, make choices, and engage in self-care, they may begin to recognise their own capacity to initiate positive changes aligned with their treatment goals.

Referring to an existing NBI within a programme, one clinician noted:

“One of the members leads the nature walking that instills confidence and novel feelings to restore once lost confidence in themselves.”

(03)

Clinicians further explained that NBIs may contribute to improved self-esteem by encouraging the development of stress management strategies and emotional regulation skills. Overall, they perceived these interventions as fostering a sense of confidence, autonomy, and psychological resilience among mental health service users.

### 3.10. Physical Fitness

Some mental health clinicians also highlighted the physical health benefits associated with NBIs. One participant remarked, “Physical well-being is not of a very high importance as compared to mental health.” (06).

Clinicians further noted that many mental health service users may experience reduced physical fitness or poorer overall physical health, partly due to sedentary lifestyles and side effects of psychotropic medications, such as weight gain. They therefore suggested that NBIs, particularly those involving walking and other forms of physical activity, could contribute to improved physical health alongside mental wellbeing, supporting a more holistic approach to recovery.

### 3.11. Perceived Barriers to NBIs Within Mental Health Services

Although clinicians strongly supported the use of NBIs for improving mental well-being, they also raised concerns regarding their successful implementation, particularly in relation to nature-based walking groups. They identified a range of potential barriers at the individual, social, and organisational levels that could affect participation and delivery. These included patient-related factors such as anxiety, low motivation, and scepticism, as well as broader contextual challenges such as gender-based limitations in accessing natural spaces and institutional safety policies that may restrict outdoor activities.

### 3.12. Individual Barriers

Mental health clinicians identified several potential barriers to participation in NBIs, including anxiety, low motivation, and scepticism toward novel nature-based approaches; reduced physical fitness; fear of group-based adjustment; transportation difficulties; and gender-related constraints, such as the need for separate male and female groups.

Clinicians expressed concern that some mental health service users may be hesitant to engage in NBIs due to doubts about their effectiveness. They noted that nature-based activities are not universally valued, and some individuals may not associate natural environments with mental health benefits. One clinician suggested that patients might underestimate the value of such interventions, stating: “What is the need for such groups, to walk in a deserted place? (09).” Others similarly anticipated that some patients would require additional encouragement and motivation to participate. For example, one clinician reported a patient response to recommendations about nature engagement: “What should I do in nature, how will it help me?” (08).

According to clinicians, resistance to participation may also stem from physical limitations, including sedentary lifestyles, fatigue, low motivation, and reluctance to engage in walking or physical activity (04). They further noted that some patients may feel physically unwell or lack the confidence required to take part in structured outdoor programmes.

Mental health symptoms were also considered a significant barrier to engagement in NBIs. Clinicians expressed concern that individuals with anxiety or phobic disorders may avoid social interaction, limiting their willingness to join group-based activities. One clinician described such reluctance as follows: “She does not want to join the walking group as she has social anxiety and fears judgment” (05). In addition, clinicians highlighted that some patients may be preoccupied with their symptoms, making it important to consider environmental stimulation levels. For instance, one participant noted: “There can be environments with low stimulation that support persons with psychosis” (07). Special consideration was also emphasised for individuals experiencing severe mental illness, particularly those with suicidal ideation or major depressive disorder. One clinician cautioned: “If you are managing a patient with suicidal tendencies, you must stay away from high range mountains, and it is better not to take them to any remote nature-based settings (10).”

Group dynamics were also identified as a potential challenge in implementing NBIs. Clinicians suggested that differences in age and physical ability could affect group cohesion and participation. For example, younger patients may feel uncomfortable joining groups with older adults due to perceived mismatches in physical pace or ability (11). One clinician elaborated:

We can walk with some people who may not be very well and others who may function well. So, younger patients may feel hesitant. Similarly, the older one will feel, “I am not as physically fit as they are, so I should not continue with the group”.

(12)

In addition, stigma surrounding mental health was seen as a barrier, with some patients feeling ashamed or uncomfortable participating in community-based activities. Feelings of shyness, social intimidation, and fear of judgment were also thought to contribute to withdrawal from group settings.

Finally, geographical and infrastructural limitations were highlighted as practical barriers. Clinicians noted that many patients do not drive or own private transport, and public transport is limited or inaccessible in certain areas. As a result, transportation challenges may significantly restrict the ability of individuals with limited financial and logistical resources to participate in NBIs.

### 3.13. Social and Organisational Barriers

Mental health clinicians also discussed barriers to implementing NBIs from an organisational perspective. They identified several concerns, including “safety issues (02),” “having the staff to see value in it (13),” and “poor understanding of the effectiveness of NBIs (08).” Overall, clinicians noted that mental health services tend to prioritise conventional, evidence-based medical models delivered in controlled clinical environments, which may limit openness to alternative approaches such as NBIs. This also reflected broader organisational uncertainty regarding the evidence base for NBIs in mental health care.

One clinician explained:

“Any novel approach will call out for being evidence-based, and some judgements arise as to why the staff themselves join the group. They are not motivated to do anything that does not save time or if nothing substantial is gained in tangible terms.”

(05)

Clinicians suggested that both staff and institutions may be sceptical of NBIs, particularly when these interventions occur outside traditional clinical settings such as offices or hospital environments, including natural spaces like mountains or parks. A key challenge, therefore, lies in clearly demonstrating and legitimising the effectiveness of NBIs as a valid therapeutic approach rather than viewing them as non-essential or supplementary activities.

Safety and risk management were also frequently raised as organisational concerns. Clinicians highlighted the need for formal risk assessments when conducting NBIs, noting potential issues such as patients being unable to complete walking activities, experiencing physical or psychological distress, or encountering behavioural or environmental risks such as falling or other unexpected incidents. Some clinicians felt that stringent safety protocols could limit the flexibility and effectiveness of NBIs.

One participant noted:

“If any intervention is scheduled outside the therapy office, it is linked with governmental scrutiny, and risk policies must be approved through medical councils since the environment cannot be controlled”.

(10)

Barriers were shaped by the interaction between clinical symptom severity, cultural norms, and resource-limited service infrastructure.

Clinicians further emphasised that the inherently risk-averse culture within mental health services may present additional challenges for implementing outdoor interventions.

Finally, gender-based considerations were also identified as an organisational barrier. In some contexts, separate walking groups for men and women may be required, which can complicate programme organisation and delivery. One clinician reported:

“A patient got angry when she knew that she may have to walk with other male group members, which is not suited to the cultural norms”.

(14)

Unlike evidence from high-income settings, implementation in the Hazara Division was constrained by gender norms, transport limitations, and risk governance structures.

## 4. Discussion

This study aimed to explore mental health clinicians’ perspectives on the advantages and barriers associated with implementing NBIs within community mental health services. Overall, findings indicated strong clinician support for NBIs, with willingness to recommend them to service users. Unless otherwise indicated, themes reflect the perspectives of all participants (*N* = 14). Findings relating to the effectiveness of NBIs in practice are based on the experiences of the five clinicians who had actively implemented NBIs, whereas findings concerning anticipated benefits, barriers, and future implementation include perspectives from clinicians without direct implementation experience.

Clinicians highlighted perceived benefits including nature-based mindfulness, stress reduction, improved social relationships, and supportive communities of nature-based activity for patients (Marselle et al., 2019). Clinicians described nature engagement as embedded in everyday life in the Hazara region and linked to emotional restoration, stress relief, and therapeutic connection-building. They noted cultural familiarity with natural environments, including “bush walking,” agroforestry, and regular interaction with forests and mountainous landscapes (14). Nature exposure was perceived as restorative, with patients describing it as “healing to remorse, stress, and negative emotions” (08) and that “it relieves stress completely” (10). Sensory engagement, such as “feeling grounded while walking on grass with bare feet” (03), noticing “wood pigeons in the trees” (04), and experiencing “the smell of conifers” (06), was viewed as calming and meditative. Clinicians also reported that nature-based settings facilitated therapeutic rapport and emotional expression, noting a “natural flow of conversation that is distinct as compared to a confined sitting space” (09) and describing successful engagement with a trauma-affected client through walks in botanical gardens and interaction with wildlife (11). Overall, NBIs were seen as enhancing emotional awareness, confidence, and social connectedness, particularly through group walking activities.

Despite these perceived benefits, clinicians identified several implementation challenges. Concerns were raised regarding patient suitability, particularly for individuals with severe mental illness, social anxiety, or suicidal ideation. One clinician cautioned against taking suicidal patients to “high range mountains” or remote natural settings due to safety concerns (10). Group-based interventions were also perceived as potentially difficult because of differences in age, physical ability, and comfort levels within mixed groups (11,12). In addition, clinicians identified institutional and sociocultural barriers, including limited staff motivation, insufficient understanding of the evidence base for NBIs, governmental risk regulations, and gender-related cultural sensitivities. For example, one clinician described resistance from a female patient who felt uncomfortable walking alongside unrelated male group members due to cultural norms (14). Collectively, these findings suggest that while NBIs were viewed as culturally meaningful and therapeutically valuable within the Hazara context, their implementation may require careful adaptation to clinical, institutional, and sociocultural realities.

Clinicians broadly perceived NBIs as potentially supportive of recovery processes for individuals experiencing mental health difficulties. These perceptions align with the Biophilia Hypothesis, which proposes an inherent human affinity for the natural world and living systems (Lomax et al., 2024). Similarly, such notions are consistent with Nature Connectedness Theory, that stronger psychological and emotional connections to nature are associated with greater well-being benefits derived from nature exposure (C. Ma et al., 2025). In this context, clinicians viewed NBIs as a potentially valuable and novel adjunct to conventional mental health treatments, particularly for conditions such as depression and anxiety. This is especially relevant given that traditional treatment approaches may not be suitable or accessible for all individuals.

Preliminary evidence suggests potential cost-effectiveness of NBIs, but findings remain inconclusive due to limited economic evaluations (Busk et al., 2022). This perspective is consistent with Salutogenic Theory and green space research, which emphasise that engagement with natural environments can strengthen an individual’s sense of coherence, enhancing comprehensibility, manageability, and meaningfulness in life (Varadarajan et al., 2025). Clinicians also suggested that NBIs may encourage patients to adopt new coping strategies for managing mental health symptoms, thereby enhancing confidence and self-efficacy in recovery processes (Kotera, 2021). In line with existing evidence, NBIs were seen as facilitating social connection and reducing feelings of isolation, as group-based nature activities may allow individuals to experience companionship without requiring intense or sustained social interaction (Leavell et al., 2019).

Nevertheless, several implementation challenges were identified. These barriers often reflected clinicians’ assumptions regarding the capabilities and needs of mental health service users. For example, clinicians noted that motivation may be particularly difficult to sustain during depressive episodes (Petrov & Semenova, 2025). Clinicians suggested that adaptations such as passive exposure to natural environments (e.g., viewing green spaces) rather than active participation in walking may be appropriate for individuals experiencing acute or severe symptoms, including suicidal ideation.

Additionally, clinicians expressed concerns regarding physical fitness, age-related differences, and gender dynamics, all of which may influence participation and group cohesion in NBIs (Kaleta et al., 2025; Odell et al., 2024).

Clinicians also suggested that resistance to NBIs may stem from their novelty, with some patients preferring familiar, traditional therapeutic approaches (Salonen et al., 2022). At an organisational level, limited awareness and understanding of NBIs among healthcare staff were identified as key barriers, potentially hindering behavioural change and the integration of NBIs into routine mental health care.

While the findings indicate perceived benefits of NBIs, these should be understood within the broader socio-political and economic context shaping mental health in the Hazara region. Poverty, limited access to healthcare, and wider structural stressors, including regional insecurity and political instability, are likely to significantly influence mental health outcomes. Accordingly, NBIs should not be viewed as standalone solutions to complex mental health needs, but rather as complementary, adjunctive interventions that may support wellbeing alongside wider structural and service-level responses.

The findings should also be interpreted within their cultural context. In the Hazara region, clinicians described nature as an integral part of everyday life through activities such as agriculture, forestry, and regular interaction with mountainous environments. This was described as differing from perceptions commonly reported in some Western contexts, where engagement with nature is often conceptualised as a recreational or leisure activity. Such cultural and environmental differences may influence perceptions of, and engagement with, NBIs and should be considered when assessing the transferability of these findings to other settings. Future research should explore how cultural understandings of nature shape the implementation and acceptability of NBIs across diverse populations.

On the contrary, some studies suggest differently. A large school-based cluster randomized-control trial found no overall depressive symptom reduction after NBI after 12 weeks, though children with higher baseline symptoms improved more (Loose et al., 2024). Workplace reviews report predominantly positive mental health effects from NBIs, but mixed findings for recovery and restoration are reported related to recovery and physiological outcomes (Gritzka et al., 2020).

### 4.1. Strengths and Limitations

The present study offers valuable insights into mental health clinicians’ perspectives on NBIs within mental health services in Hazara Division, Khyber Pakhtunkhwa, Pakistan. Exploring these perspectives is particularly important, as the successful adoption and integration of NBIs is likely to depend on clinicians’ preparedness, confidence, and organisational support. Eligible participants were aged 18 years or older and currently employed in a role involving direct mental health support within the Hazara Division. Direct mental health support was defined as providing assessment, treatment, rehabilitation, or ongoing psychosocial support to individuals with mental health conditions. This included psychiatrists, psychologists, mental health nurses, social workers, peer support workers, and community mental health workers. Administrative personnel and professionals without direct mental healthcare responsibilities were excluded. Furthermore, examining clinicians’ own relationships with nature provided deeper insight into how personal experiences and motivations may shape their attitudes toward NBIs.

Despite these strengths, several limitations should be acknowledged. The study was conducted within a single local health district characterised by rich natural environments and relatively easy access to green spaces, which may limit the transferability of findings to more urbanised or resource-constrained settings. However, it is important to note that urban areas can still support NBIs through accessible green spaces such as public parks, community gardens, and botanical gardens. Another limitation is that the study focused exclusively on clinicians’ perspectives, without including the views of mental health service users. Future research is planned to address this gap by exploring consumers’ experiences, perceptions, and interest in NBIs. In addition, participant self-selection may have introduced bias, as clinicians with a stronger personal affinity for nature may have been more likely to participate. A further limitation is that approximately 85% of participants were female. Although the study aimed to explore clinicians’ perspectives rather than compare experiences across gender, the predominance of female participants may have influenced the range of views captured regarding NBIs. Future qualitative research should seek greater gender diversity to explore whether perceptions of NBIs differ across clinicians and to enhance the transferability of the findings.

The first author’s regional familiarity likely facilitated rapport and culturally sensitive data collection but may also have shaped participant responses and interpretation; reflexive practices were therefore maintained throughout the study to mitigate this.

Member checking was not conducted due to logistical constraints, which may affect confirmability. However, credibility was supported through verbatim transcription, systematic coding, and peer debriefing. Recruitment flow and non-participation were not systematically recorded, limiting assessment of potential selection bias.

Despite these limitations, the study provides important insights into clinicians’ perspectives on NBIs in a resource-constrained and culturally specific setting.

Lastly, this study may pose the risk of selection bias arising from the use of snowball sampling. Clinicians with greater interest in or exposure to NBIs may have been more likely to participate, which may have influenced the range of perspectives captured. Therefore, while the study provides rich contextual insights, the transferability of findings to other settings should be considered with caution. However, in line with qualitative methodology, transferability is supported through detailed contextual description rather than statistical generalisation. Nevertheless, despite these limitations, the study provides meaningful contributions to the development of mental health service delivery and future research on NBIs. It is important to take into consideration that the relationship between nature exposure and mental health should be interpreted within broader structural and contextual conditions. In regions affected by chronic socioeconomic adversity, political instability, and limited healthcare access, these factors may substantially shape mental health outcomes and influence the extent to which NBIs are beneficial. Therefore, these contextual conditions should be considered when interpreting the transferability of findings, and future research should further examine the role of such structural determinants.

### 4.2. Implications

The findings suggest that the implementation of NBIs in Hazara Division, Khyber Pakhtunkhwa, Pakistan, may depend substantially on both organisational capacity and systemic readiness to integrate and sustain recovery-oriented models of care. This includes not only conceptual alignment with recovery-focused approaches but also practical considerations such as workload distribution, staffing, and service organisation across teams. The study also highlights that scepticism and limited awareness of NBIs within both communities and health systems (Burrell et al., 2024) may hinder adoption and therefore require targeted awareness-raising initiatives, potentially led by governmental and public health organisations. In this regard, collaboration between environmental stakeholders and healthcare professionals may be important for supporting the successful integration of NBIs into mental health services. Early involvement of patients in programme design and planning may further enhance acceptability and engagement.

Future research should prioritise the development of a stronger evidence base for NBIs within mental health care. In particular, well-designed clinical trials are needed to evaluate the effectiveness of group-based NBIs and to better understand their outcomes. Further studies should also explore service users’ experiences, perceptions, and preferences regarding NBIs to ensure interventions are person-centred and contextually appropriate. Additionally, research examining the adaptability and effectiveness of NBIs across both urban and rural settings is needed, with particular attention to how environmental and infrastructural differences influence implementation and outcomes.

From a policy and practice perspective, the findings suggest that successful implementation of NBIs will require organisational and system-level support. Mental health services could consider incorporating NBIs as complementary interventions within existing recovery-oriented care pathways, supported by appropriate clinical guidance, staff training, and interdisciplinary collaboration. Policymakers may also consider investing in accessible green spaces, strengthening partnerships between healthcare providers, local authorities, and environmental organisations, and developing referral pathways that facilitate safe and equitable access to nature-based programmes (Pauleit et al., 2021).

## 5. Conclusions

Increasing global pressures on mental health systems underscore the need to explore innovative and complementary approaches to care delivery. NBIs represent a promising avenue in this regard, and the findings of this study indicate that mental health clinicians are broadly supportive of their integration into routine services. Clinicians demonstrated strong belief in the therapeutic and restorative value of natural environments, with many also drawing on their own positive experiences of nature to inform their clinical perspectives and recommendations to service users.

In combination with the growing empirical evidence linking exposure to nature with improved psychological well-being, these findings point to meaningful potential for embedding NBIs within standard mental health care pathways. Such integration may help strengthen existing models of care by offering accessible, cost-effective, and person-centred adjuncts to conventional treatment. Ultimately, the incorporation of NBIs into mental health services may contribute to improved recovery outcomes and enhanced well-being among individuals accessing care. Moreover, future research with larger, profession-specific samples could explore whether attitudes towards NBIs differ across clinical disciplines.

## Figures and Tables

**Table 1:** Demographic and Professional Characteristics of the Participants (*N* = 14).

Characteristic	Description
Total participants	14 mental health clinicians
Professional backgrounds	Psychology, psychiatry, nursing, peer support, social support, community outreach services
Age range	26–62
Mean age	42.5 years
Gender	11 females, 03 males
Ethnicity	Diverse ethnic backgrounds
Professional experience range	2–35 years
Mean professional experience	14.2 years

## Data Availability

The anonymised interview transcripts from this study are publicly available in Figshare https://figshare.com/s/a0b0ffd9ed076987070c (accessed on 20 June 2026). Participants are identified using numerical codes (P01–P14), with all identifying information removed to ensure confidentiality and minimise the risk of deductive disclosure. All transcripts were fully anonymised prior to deposition in accordance with ethical approval requirements.

## Supplementary Materials

The following supporting information can be downloaded at: https://www.mdpi.com/article/10.3390/bs16081298/s1, File S1: Interview Guide For Mental Health Clinicians in Hazara Division. File S2: COREQ 32-item Checklist (Qualitative Research Reporting).

## Abbreviations

The following abbreviations are used in this manuscript:

NBIs	Nature-Based Interventions
LMIC	Low- and Middle-Income Countries
