# Supplementary material for: Clinicians’ Perceptions of Nature-Based Interventions in Mental Health Care: A Qualitative Descriptive Study from Pakistan
Source: Behav Sci (Basel). 2026 Jul 30;16(8):1298. doi: 10.3390/bs16081298 (PMC13510135; doi:10.3390/bs16081298)
Supplement: Supplementary file 1 [file behavsci-16-01298-s001.zip › behavsci-4394297-supplementary.pdf]

File S1: INTERVIEW GUIDE FOR MENTAL HEALTH CLINICIANS IN HAZARA DIVISION

Section A: Clinicians' views and current practice

1. What is your overall opinion of nature-based interventions in mental health care in Hazara?
2. Do you personally encourage or recommend nature-based activities to your patients? Why or why not?
3. How do your own experiences or views of nature influence your clinical practice (if at all)?

Section B: Potential benefits

4. What potential benefits do you think patients may experience from engaging in nature-based activities s?
5. How might NBIs affect patients' stress levels, emotions, or mental health symptoms?
6. Do you think NBIs can influence patients' social relationships or sense of empowerment? If so, how?
7. Are there any physical health or lifestyle benefits you associate with NBIs?
8. In what ways might NBIs support recovery or complement existing treatments?

Section C: Challenges

9. What challenges might limit the use of NBIs in your clinical setting?
10. Are there any patient-related factors (e.g., motivation, anxiety, beliefs) that may affect participation?
11. Are there organisational or service-level barriers (e.g., resources, policies, staffing- and keeping in mind socio-cultural/socio-political aspects)?
12. Are there any cultural, gender-related, or environmental factors that may restrict access to nature-based activities?
13. Do you foresee any safety or logistical concerns with implementing NBIs?

Section D: Feasibility

14. How feasible do you think it is to integrate NBIs into routine mental health services in your setting?
15. What support or changes would be needed to implement NBIs effectively?

Section E: Final verdict

16. Do you think NBIs should be formally included in mental health care? Why or why not?
17. Do you have any additional comments or suggestions?

File S2: COREQ 32-item Checklist (Qualitative Research Reporting)

Domain 1: Research team and reflexivity

Item No.	Item	Guide questions/description	Reported in study
1	Interviewer/facilitator	Which author conducted the interviews?	The first author conducted all interviews
2	Credentials	What were the researcher's academic qualifications?	Doctoral student in Psychology, now is qualified with PhD in psychology as of 10th June 2026

3	Occupation	What was the researcher's occupation at the time?	Doctoral research student
4	Gender	Was the researcher male or female?	Female
5	Experience and training	What experience/training did the researcher have?	Trained in qualitative research methods and supervised by senior academics
6	Relationship with participants	Was there any prior relationship?	No prior relationship with participants
7	Participant knowledge of interviewer	What did participants know about the researcher?	Participants were informed of the academic purpose of the study
8	Interviewer characteristics/reflexivity	Was reflexivity considered?	Reflexivity was maintained through team discussions and supervision

Domain 2: Study design

Item No.	Item	Guide questions/description	Reported in study
9	Methodological orientation	What methodology guided the study?	Qualitative descriptive design (Sandelowski, 2000)
10	Sampling method	How were participants selected?	Purposive and snowball sampling
11	Sample size	How many participants?	14 mental health clinicians
12	Non-participation	Was refusal or dropout reported?	No refusals reported
13	Setting of data collection	Where were interviews conducted?	Naturalistic settings convenient to participants
14	Presence of non-participants	Anyone else present during interviews?	None reported
15	Description of sample	How is sample described?	Clinicians from diverse mental health disciplines
16	Interview guide	Was it pilot tested or developed?	Semi-structured guide developed by research team
17	Repeat interviews	Were follow-up interviews conducted?	No repeat interviews

18	Audio/visual recording	Were interviews recorded?	Audio-recorded
19	Field notes	Were field notes taken?	Yes, immediately after interviews
20	Duration	Interview length	20–68 minutes (mean 35 minutes)
21	Data saturation	Was saturation discussed?	Data collection stopped when no new themes emerged

---

Domain 3: Analysis and findings

Item No.	Item	Guide questions/description	Reported in study
22	Transcripts returned	Were transcripts returned to participants?	No
23	Data coding	How were data coded?	Inductive coding using Braun & Clarke (2019) framework
24	Number of coders	Who coded the data?	First author; reviewed second and third authors.
25	Coding tree	Was a coding framework developed?	Yes, themes and subthemes generated

26	Theme derivation	Were themes inductive or deductive?	Inductive (data-driven)
27	Software	What software was used?	HyperRESEARCH 4.5.7
28	Participant checking	Was member checking done?	No
29	Quotations presented	Are participant quotes included?	Yes, illustrative quotations included
30	Data consistency	Was consistency checked?	Yes, through peer debriefing and supervision
31	Major themes clarity	Are major themes clearly defined?	Yes
32	Minor themes clarity	Are minor themes reported?	Yes (where applicable)
